# Recommendations for antibacterial prophylaxis in children receiving chemotherapy: a joint initiative of SITIP and infectious disease group of AIEOP

**DOI:** 10.1186/s13052-025-02141-1

**Published:** 2025-11-25

**Authors:** Daniele Zama, Davide Leardini, Francesco Baccelli, Edoardo Muratore, Elio Castagnola, Margherita Del Bene, Maia De Luca, Elisa Funiciello, Federica Galaverna, Riccardo Masetti, Paola Muggeo, Rosa Maria Mura, Katia Perruccio, Erica Ricci, Manuela Spadea, Andrea Lo Vecchio, Simone Cesaro

**Affiliations:** 1 Pediatric Emergency Unit, IRCCS Azienda Ospedaliero-Universitaria di Bologna, Bologna, Italy; 2 Pediatric Hematology and Oncology, IRCCS Azienda Ospedaliero-Universitaria di Bologna, Bologna, Italy; 3 Pediatric Infectious Diseases Unit, IRCCS Istituto Giannina Gaslini, Genova, Italy; 4 Pediatric Infectious Disease Unit, Department of Maternal and Child Health, University Hospital “Federico II”, Napoli, Italy; 5 Infectious Disease Unit, Bambino Gesù Children’s Hospital, IRCCS, Roma, Italy; 6 Pediatric Infectious Diseases Unit, Regina Margherita Children’s Hospital, University of Turin, Torino, Italy; 7 Department of Pediatric Hematology and Oncology, University Hospital of Policlinico of Bari, Bari, Italy; 8 Pediatric Oncology Unit, Azienda Ospedaliera Brotzu, Cagliari, Italy; 9 Pediatric Oncology Hematology, Mother and Child Health Department, Santa Maria della Misericordia Hospital, Perugia, Italy; 10 Pediatric Oncohematology, Stem Cell Transplantation and Cell Therapy Division, Regina Margherita Children’s Hospital, Torino, Italy; 11 University of Turin, Torino, Italy; 12 Pediatric Hematology Oncology Unit, Department of Mother and Child, Azienda Ospedaliera Universitaria Integrata, Verona, Italy

**Keywords:** Antibiotic prophylaxis, Pediatric cancer, Chemotherapy, Neutropenia, Multi-drug-resistant bacteria

## Abstract

**Background:**

Current guidelines for managing infections in pediatric patients with cancer do not recommend routine antibiotic prophylaxis (AP). However, several aspects of AP, including the role of diagnosis, the impact of neutropenia duration, screening for resistant bacterial colonization, and antibiotic stewardship, remain a matter of debate.

**Methods:**

To address these issues, a panel of experts from the Italian Association of Pediatric Hematology and Oncology (AIEOP) and the Italian Society of Pediatric Infectious Diseases (SITIP) conducted a Delphi consensus. A comprehensive literature review and a national survey of pediatric oncology centers identified clinically relevant topics that are not fully covered by current guidelines. Based on this, the expert panel developed and voted on 14 statements covering eight key areas: the role of diagnosis, duration of neutropenia, screening for colonization with antibiotic resistant bacteria, use of validated risk scores, implementation of antimicrobial stewardship programs, periodic monitoring of local epidemiology, choice of antibiotic for prophylaxis, and the risk of resistance following prophylaxis.

**Results:**

The panel reached a consensus against prophylaxis in patients receiving monoclonal antibody therapy and advised against using the duration of neutropenia alone as a criterion to initiate prophylaxis, recommending it only for severe neutropenia (< 500/mm³). They also emphasized the importance of screening for multidrug resistant bacteria and implementing antimicrobial stewardship supported by specialist consultation.

**Conclusions:**

These recommendations provide guidance for clinicians on the selective use of AP, supporting informed decision making while ensuring appropriate treatment and reducing the emergence of multidrug resistant bacterial infections.

**Supplementary Information:**

The online version contains supplementary material available at 10.1186/s13052-025-02141-1.

## Introduction

Children receiving chemotherapy for oncological diseases are at high risk of bacterial infections [1]. The associated morbidity and mortality have prompted several strategies to reduce their burden in this setting [2, 3]. Systemic antibiotic prophylaxis (AP) is one of the approaches used to prevent bacterial infections and febrile neutropenia (FN), but its use may be limited by drug-related toxicity and the emergence of antibiotic resistance [4]. Based on these concerns and on evidence from the literature showing no significant reduction in overall mortality associated with AP, the 8th European Conference on Infections in Leukemia (ECIL-8) issued a strong recommendation against routine antibacterial prophylaxis, even in higher-risk settings, although the quality of evidence was low. However, ECIL-8 emphasized that a careful risk–benefit evaluation may still favor antibacterial prophylaxis in selected patients, depending on specific circumstances [4]. In contrast, the Infectious Diseases Society of America (IDSA) guidelines [5] recommend considering systemic antibacterial prophylaxis in children with acute myeloid leukemia (AML) and relapsed acute lymphoblastic leukemia (ALL) receiving intensive chemotherapy expected to result in severe neutropenia (absolute neutrophil count [ANC] < 500/µL) lasting at least 7 days, with a weak recommendation but high-quality evidence [5]. Restricting AP to those at highest risk of infection may help limit antibiotic use while preserving clinical benefit, although precise risk factors to identify patients most likely to benefit are still lacking. To provide guidance for clinicians on whether AP is appropriate and in which patients it should be considered, the Italian Association of Pediatric Hematology and Oncology (AIEOP) and the Italian Society of Pediatric Infectious Diseases (SITIP) developed shared recommendations for AP in pediatric patients undergoing chemotherapy for oncological diseases.

## Methods

To develop the recommendations, a modified Delphi consensus process was used [6]. Details of the process are provided in the Supplementary Methods.

## Results

### Delphi process

**Fig. 1:**
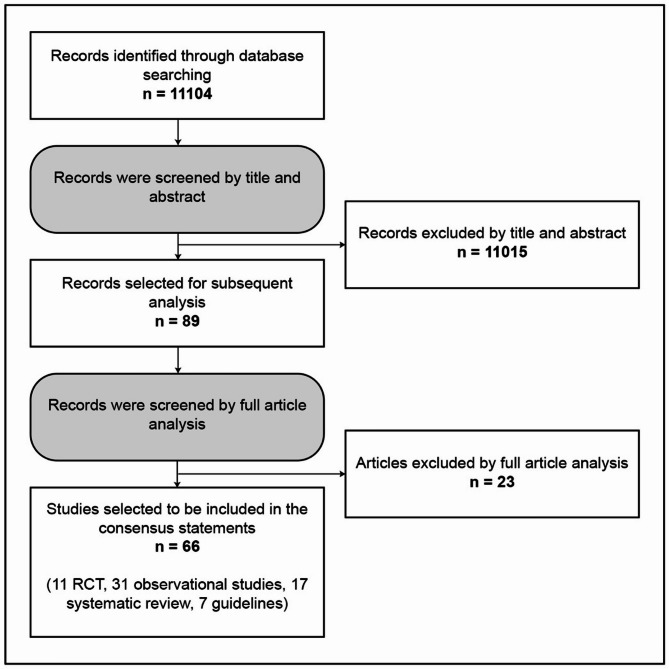
Diagram depicting the process of literature search and selection

The literature search of peer-reviewed articles published in English from January 1, 2012, to December 31, 2022, identified 66 studies that were included in the consensus process (Fig. 1). The expert committee defined eight areas of interest and produced 14 statements focusing on these areas. After the first round of voting, consensus was reached on all statements, and no further modifications were required. The statements and detailed voting results are presented in Table 1, with supporting evidence provided below and in Supplementary Table 1.

**Table 1:** Summary of the recommendations. *1.3 & 1.4: although the expert panel separated statements 1.3 and 1.4, from 1.1 and 1.2 for greater clarity, their explanation is included in the text of points 1.1 and 1.2, given that the research in the literature was based on the role of diagnosis

No.	Statement	Consensus median vote
1.What is the role of the underlying oncological diagnosis in the adoption of AP for FN?		
1.1	AP is not recommended in pediatric patients undergoing induction or reinduction chemotherapy for standard-intermediate risk ALL.	**9.0**
1.2	AP may be an option in pediatric patients undergoing treatment for high-risk ALL, relapsed ALL and AML, but the periodic surveillance of the type of bacterial infection and the rate of antibiotic resistance is strongly recommended.	**8.9**
1.3^*^	AP in patients treated as monotherapy with target monoclonal antibodies such as blinatumomab, inotuzumab-ozogamicin gentuzumab/ozogamicin, in absence of specific patient’s risk factors (mucositis, neutropenia) is not recommended.	**9.7**
1.4^*^	AP in patients undergoing chemotherapy for solid tumors is generally not recommended.	**9.6**
2.Does the expected duration and depth of neutropenia influence the choice to perform AP?		
2.1	The expected duration and depth of neutropenia should not be considered as the main criteria for the choice to perform AP.	**8.2**
2.2	In the selected situation in which the antibacterial prophylaxis is administered, it is advisable to restrict the duration of prophylaxis to severe neutropenia (ANC < 500/µL) and discontinue the prophylaxis at the time of confirmed myeloid recovery.	**8.5**
3.What is the role of screening for colonization by multi-drug resistant bacteria conducted before the start of chemotherapy in the decision to perform AP?		
3.1	Screening for colonization by antibiotic-resistant bacteria [e.g. extended-spectrum beta-lattamases (ESBL), carbapenem-resistant *Enterobacteriales* (CRE), vancomycin-resistant Enterococcus (VRE), methicillin-resistant *S. aureus* (MRSA)] play a key role to prevent the hospital diffusion of these germs (patient’s isolation and personnel contact precautions), to set the correct empiric antibiotic therapy in case of febrile neutropenia, and to adopt decolonization procedures, if indicated.	**9.6**
4.What is the role of validated scores predictive of NF in the choice of starting AP in children with cancer?		
4.1	The choice of AP by the application of scores predicting the risk of bacterial infections before the onset of neutropenia, is not supported by sufficient data.	**9.1**
4.2	However, the use of a validated or an internal score to select which patients may benefit from AP is suggested.	**7.6**
5.Is there a role for antibacterial stewardship programs and how should antimicrobial stewardship programs be applied in the decision to perform AP?		
5.1	Antimicrobial stewardship programs, aimed at identifying those selected pediatric oncology patients with an unbalanced benefit/risk ratio, who potentially could benefit from an AP are warranted.	**8.9**
5.2	Consultation with an infectious disease specialist regarding AP is recommended.	**9.3**
6.What is the role of periodic assessment of local ecology in the choice of prophylactic antibiotic therapy?		
6.1	A periodical monitoring of local rates of antimicrobial resistance to early identify the emergence of specific strains or species and discover changes in the antimicrobial susceptibility profile of the organisms is recommended.	**9.9**
7.Which agents should be used for systemic AP?		
7.1	When AP is considered to reduce the incidence of infections, the expert panel suggest levofloxacin, after a careful risk-benefit evaluation.	**8.6**
8.Does systematic use of AP increase the risk of developing infections from antibiotic-resistant germs?		
8.1	AP may increase the rate of antibiotic resistance and the risk of BSI caused by pathogens resistant to the ongoing prophylaxis or by MDR bacteria and thus should be carefully evaluated.	**9.6**

### Supporting evidence

#### Evidence for 1.1

One study conducted in a low-income country failed to demonstrate any benefit of ciprofloxacin prophylaxis [7]. In this trial, several factors related to the local context may have influenced the results, including a high proportion of malnourished children that were not balanced during randomization, a very high rate of toxic deaths (23% compared with 2–4% in high- and middle-income country settings), and a significant rate of treatment abandonment (35%) [7]. In a second randomized controlled trial, ciprofloxacin prophylaxis was effective in reducing febrile episodes during induction (− 23.7%; 95% CI, − 45.6% to − 1.8%) but not during consolidation (9.8%; 95% CI, − 17.8% to 37.5%). This finding can be explained by the markedly different proportions of patients with neutropenia: during induction, 43.7% were neutropenic, with a median ANC of 45 cells/mm³ (IQR 0–515), whereas during consolidation only 4.4% were neutropenic, with a median ANC of 1180 cells/mm³ (IQR 576–1960; *p* < 0.05) [8]. Despite the positive effect of ciprofloxacin prophylaxis on febrile episodes, the study had several limitations: the small sample size (*n* = 95, of whom 71 had ALL), the very low prevalence of bacteremia in the control group (2%), which does not reflect the risk usually associated with contemporary ALL induction regimens (known to exceed 10%), and the absence of stratification by ALL risk group, which prevented assessment of prophylaxis efficacy in relation to treatment intensity. One single-center observational study showed that levofloxacin prophylaxis reduced the risk of FN by more than 70% (adjusted OR 0.23, 95% CI 0.14–0.40; *p* < 0.001) in pediatric patients with ALL during induction. The main drawbacks of this study were its retrospective design, the single-center setting (which increases the likelihood of confounders and limits generalizability), the long period over which it was conducted, and the small sample size, which was inadequate to assess the medium- or long-term risk of antibiotic resistance [9]. Two systematic reviews that included the above-mentioned studies confirmed the benefit of AP in this population, demonstrating reductions in the incidence of febrile episodes, clinically documented infections, microbiologically documented infections, bacteremia, hospital admissions, length of stay, and fever duration. However, the long-term impact of prophylaxis on antimicrobial selective pressure remains an open question [10, 11].

#### Evidence for 1.2, 1.3, 1.4

 In the study by Alexander et al., which included 200 patients with AML or relapsed ALL, levofloxacin prophylaxis administered during two consecutive chemotherapy cycles significantly reduced the risk of bacteremia (risk difference − 21.6%; 95% CI, 8.8% to 34.4%; *p* = 0.001) and FN (risk difference − 10.8%; 95% CI, 4.2% to 17.5%; *p* = 0.002) compared with placebo [12]. No differences were observed in the incidence of severe infections, invasive fungal disease, or *C. difficile*–associated diarrhea [12]. No musculoskeletal toxic effects were reported in the levofloxacin group. Although the number of isolates was too small for statistical analysis, a high proportion of levofloxacin-resistant pathogens was detected in bacteremia episodes among patients receiving prophylaxis. Moreover, no data are available on the long-term effects of levofloxacin on antimicrobial selective pressure. All five systematic reviews evaluated the efficacy of different AP regimens in patients with hematological malignancies (both adults and children), but their results were not consistent [10, 11, 13–15]. Owattanapanich et al. reported significantly lower rates of FN, bacteremia, and microbiologically documented infections, including C. difficile–associated diarrhea, in patients who received levofloxacin prophylaxis, although mortality was similar between groups [10]. Likewise, Egan et al. concluded that fluoroquinolone (FQ), trimethoprim-sulfamethoxazole, and cephalosporin prophylaxis reduced bacteremia but did not significantly affect overall mortality [13]. In contrast, Gafter-Gvili et al. found that AP in afebrile neutropenic patients significantly reduced all-cause mortality, with the greatest effect observed in trials evaluating quinolone prophylaxis [11]. A potential source of bias in this review was the inclusion of studies combining FQ with other antibiotics as part of the intervention. Importantly, none of the available studies evaluated the long-term effectiveness, adverse effects, or resistance outcomes associated with routine prophylaxis, leaving a major knowledge gap. No studies have assessed the use of gemtuzumab-ozogamicin in pediatric patients. However, the most recent ECIL-9 clinical practice guidelines report no specific risk of infection or FN associated with gemtuzumab ozogamicin monotherapy in adult patients [16].

#### Evidence for 2.1

 The selected studies did not account for differences in severity or depth of neutropenia when defining patient eligibility for antibacterial prophylaxis. Moreover, standard AP carries potential risks, including increased antimicrobial resistance [12, 17] and *C. difficile* infection [12, 17], while evidence for a significant reduction in infection-related mortality remains limited [8, 12]. The prospective multicenter SPOG 2015 FN study suggested that a score based on the expected duration of severe neutropenia could help predict the risk of FN with safety-relevant events (bacteremia, severe sepsis, intensive care unit admission, death). This approach would allow restriction of prophylaxis to patients at highest risk of bacterial infections with complications [17]. In pediatric patients with solid tumors receiving chemotherapy of varying intensity, Saito et al. [18] concluded that a white blood cell count below 100/µL during neutropenic periods may represent a useful criterion to guide consideration of prophylactic antibiotics.

#### Evidence for 2.2

 Recently published guidelines in pediatric cancer and hematopoietic stem cell transplantation [5] define severe neutropenia as an ANC < 500/µL. When severe neutropenia is expected to last more than 7 days, a careful evaluation of patient history and local epidemiology is required. In pediatric patients, one randomized controlled trial [12], several prospective observational studies [19, 20], and a retrospective study [18] investigated different neutropenia thresholds, ranging from while blood cells < 1000/µL [8] to ANC < 500/µL [21] or ANC < 100/µL in more recent studies [5, 12]. AP, once initiated, should be discontinued at neutrophil recovery. The threshold for neutropenia recovery and discontinuation of prophylaxis was defined as ANC >200/µL in two prospective studies [19, 20] and one randomized controlled trial [12]. It appears reasonable to withdraw prophylaxis when ANC exceeds 200/µL and is rising after nadir, in the context of clear bone marrow recovery.

#### Evidence for 3.1

 No study has been conducted to determine which agent should be used for AP based on colonization status. Moreover, most studies enrolled patients only once [22] and data on repeated prophylactic courses are very limited [12], with scarce evidence on the development of antibiotic resistance. Epidemiological studies highlight an increased rate of resistant strains in patients receiving AP [8, 23, 24]. Laoprasopwattana et al. reported reduced susceptibility of *E. coli* and *K. pneumoniae* to ciprofloxacin after just one week in the prophylaxis group, underscoring the need for long-term studies on the intestinal flora [24]. Similarly, Tunyapanit et al. [24] described reduced susceptibility to agents other than those used in AP, by evaluating the antibiotic sensitivity of colonizing strains obtained from serial rectal swabs during ciprofloxacin prophylaxis. MIC50 values for ceftazidime in intestinal microflora significantly increased from baseline to subsequent weekly swabs in the ciprofloxacin group (*p* < 0.01). Previous antibiotic exposure, whether as prophylaxis or therapy, is significantly associated with antibiotic resistance in Gram-negative bacteremia [25]. Close monitoring is therefore recommended to detect resistant strains, particularly CRE, VRE, or MRSA. In cases of colonization, strict isolation measures are essential to prevent in-hospital spread [26, 27]. For MRSA colonization, decolonization measures have been shown to achieve successful and lasting eradication in 76.5% of patients [28]. In contrast, decontamination in cases of colonization by other pathogens (e.g., Gram-negative bacteria) has not proven effective in the long term. Colonization screening should be performed periodically. As emphasized in the ECIL guidelines, screening results should guide the adjustment of empirical treatment according to resistance profiles in patients who are colonized or have a history of infection with resistant Gram-negative bacteria [4].

#### Evidence for 4.1, 4.2

 No study has directly addressed the role of any score in predicting the risk of developing FN, bloodstream infections (BSI), or severe bacterial infections in either children or adults with cancer. Therefore, it is not currently possible to recommend a validated score to guide the decision to initiate AP. However, the validated scores included in the review were generally effective in predicting outcomes. In particular, the SPOG 2003 FN study developed two scores using a multivariate logistic regression model to predict adverse events, such as serious medical complications secondary to severe infections or bacteremia in pediatric patients with cancer presenting with FN. These scores were based on routinely available patient characteristics and effectively identified patients at higher risk of adverse events or bacteremia [29, 30]. The PICCNIC collaboration developed another accurate risk-prediction model for microbiologically defined infections in children with FN [31]. Another score, which combined clinical presentation, laboratory findings, and underlying diagnosis, predicted the risk of invasive bacterial infection, sepsis, or clinical complications in over 300 pediatric patients with FN [32]. In a report of more than 1000 FN episodes in children, an absolute monocyte count greater than 155 cells/mm³ was associated with a lower risk of bacteremia [33]. The Esbenshade Vanderbilt (EsVan) risk prediction models, a publicly available web-based score, accurately predicted the risk of BSI and reduced unnecessary antibiotic use in non-severely neutropenic pediatric patients [34]. No study has investigated the use of a clinical score prior to the onset of fever in children with cancer to predict the risk of developing FN or severe infection. Additionally, some of the aforementioned scores were particularly effective in predicting outcomes at reassessment, after 8 to 24 h of inpatient therapy for FN episodes [30, 32]. Despite the demonstrated accuracy of these scores in predicting FN outcomes, the expert panel emphasizes the need for future studies to apply these approaches in patients with cancer prior to the onset of FN. Such studies could facilitate the stratification of patients at particularly high risk of bacterial infections who might benefit from antibacterial prophylaxis.

#### Evidence for 5.1, 5.2

 No study has directly assessed the impact of antimicrobial stewardship programs (ASP) or related initiatives on AP in pediatric cancer patients. Evidence on the implementation of ASPs for general antibiotic use in pediatric and adult oncology suggests improvements in treatment appropriateness, reductions in antibiotic-related side effects, and no difference in infection-related mortality [35, 36]. Only one single-center observational study, conducted before and after the introduction of a center-specific ASP, reported recommendations regarding AP. The study found no differences in average length of hospitalization or mortality, while the total cost of antimicrobial agents decreased by 27%. However, no separate analysis of the impact of the ASP on AP was provided [37]. The potential for antibiotic resistance induced by prophylaxis should be considered in the context of local microbial ecology [38]. Prior authorization offers benefits by directly reducing unnecessary antibiotic initiation, optimizing antibiotic selection, and promoting infectious disease consultations [39]. Although prior authorization may potentially delay antibiotic administration compared with other strategies, in this context the advantages are believed to outweigh this limitation.

#### Evidence for 6.1

 The systematic review and meta-analysis by Mikulska et al., published in 2017, evaluated both pediatric and adult cancer patients receiving FQ prophylaxis [14]. Eleven randomized controlled trials comparing patients receiving FQ prophylaxis with those receiving placebo or no treatment were included in the meta-regression model. Background resistance rates to FQ varied from less than 1% to 20% in community settings and from 1% to 28% in hospital settings. The meta-regression model found no evidence that background FQ resistance influenced the efficacy of prophylaxis in reducing overall mortality, BSI, or FN, regardless of whether patients were in community or hospital settings. Although data are insufficient to recommend adjusting AP based on local resistance rates, the panel emphasizes the importance of periodic monitoring to detect the emergence and spread of specific strains or species not covered by current prophylaxis, as this could affect patient outcomes.

#### Evidence for 7.1

All three randomized controlled trials (RCTs) included in this systematic review tested the efficacy of FQ using different strategies. In the study by Alexander et al., which included 200 patients with AML or relapsed ALL in Canada, levofloxacin prophylaxis administered during two consecutive chemotherapy cycles reduced the likelihood of bacteremia (risk difference − 21.6%; 95% CI, 8.8% to 34.4%; *p* = 0.001) and FN (risk difference − 10.8%; 95% CI, 4.2% to 17.5%; *p* = 0.002) compared with placebo [12]. No differences were observed in the incidence of severe infections, invasive fungal disease, C. difficile–associated diarrhea, or musculoskeletal toxic effects [12]. In a more recent study by Dufrayer et al. (*n* = 20), levofloxacin administered from day three of induction until ANC reached 500/µL or higher was shown to be safe in children with de novo ALL and was not associated with increased colonization by carbapenemase-producing Enterobacteriaceae (CPE) or C. difficile–associated diarrhea. The rate of FN did not differ significantly between groups, likely due to the small sample size [40]. A third RCT (*n* = 95) allocated children with ALL or lymphoma to receive ciprofloxacin prophylaxis within five days of starting chemotherapy until ANC reached 1000/µL. AP significantly reduced febrile episodes in patients with ALL during induction (risk difference − 23.0%; 95% CI − 45.0% to − 0.9%; *p* = 0.046), but not during consolidation or in patients with lymphoma. Adverse effects did not differ between groups [8]. Observational studies included two designs: those comparing infection rates in patients receiving AP with historical cohorts (classic design), and those comparing infection rates before and after discontinuation of AP (discontinuation design). Among these studies, four reported results consistent with FQ efficacy observed in RCTs. These findings were also confirmed by a meta-analysis by Leardini et al. [41] Other observational studies tested different antibiotics, such as vancomycin alone or in combination, cephalosporins alone or in combination, ciprofloxacin with penicillin or teicoplanin, and piperacillin. Due to a high risk of bias, these studies do not support formal recommendations. Across all studies included in this review, AP did not demonstrate a reduction in mortality. All studies, except one [40], are included in systematic reviews and current guidelines. Ongoing surveillance and susceptibility testing remain essential, as levofloxacin prophylaxis in high-risk children has been associated with reduced bacterial susceptibility and increased minimum inhibitory concentrations to FQ, as shown in a recent study [42].

#### Evidence for 8.1

 The meta-analysis by Mikulska et al., published in 2017, evaluated the impact of FQ prophylaxis on the rate of FQ-resistant BSI across two RCTs, 14 observational studies, and one meta-analysis. Only three observational studies, two from the same center, reported an increase in FQ-resistant infections during prophylaxis, primarily in Gram-negative BSI [14]. Overall, the authors concluded that FQ prophylaxis did not increase the rate of infections caused by FQ-resistant strains, with a reported rate of 4% in both patients receiving and not receiving prophylaxis. The systematic review and meta-analysis by Leardini et al. focused on children and young adults (age >30 days and < 23 years) receiving chemotherapy for acute leukemia and receiving quinolone-based AP compared with no prophylaxis [41]. Among six studies, three reported an increased incidence of Gram-negative bacteria resistant to FQ in intestinal flora isolated from blood in patients receiving FQ prophylaxis. Similarly, Margolis et al. found that the prevalence of topoisomerase point mutations, which confer FQ resistance, increased during induction chemotherapy for ALL in participants receiving levofloxacin but not in those without prophylaxis. In contrast, two studies by Yeh et al. reported a significant reduction in ciprofloxacin and amikacin resistance among the most common Gram-negative bacilli during prophylaxis, accompanied by an increase in resistance to cefuroxime and imipenem. Data on the risk of spreading multi-drug resistant (MDR) and ESBL-producing pathogens in patients receiving FQ prophylaxis are conflicting. In the meta-analysis by Mikulska et al., eight observational studies were analyzed; in most, no increase in MDR infections was observed, while three studies reported an increase in infections caused by vancomycin-resistant enterococci or ESBL-producing Gram-negative rods [14]. No data are available on the impact of FQ prophylaxis in patients already colonized by MDR bacteria. In this subgroup, continuous antibiotic exposure could reduce the number of susceptible pathogens and promote the selection and growth of MDR strains in the intestinal flora, increasing the risk of subsequent MDR infections or horizontal transfer to other patients, potentially affecting local epidemiology [9, 20, 43].

## Discussion

Panel discussions emphasized the need to balance the benefits of AP in reducing BSI and FN against the risks, including increased antibiotic resistance, *Clostridioides difficile* infection, and disruption of the gut microbiome [44]. These considerations are consistent with international panels, despite differing conclusions [4]. Careful patient evaluation is essential when considering AP [45]. High-risk and relapsed ALL and AML patients are identified as key candidates due to their elevated infection risk (*Recommendation 1.2*), supported by randomized trials demonstrating reduced BSI and FN in pediatric AML and relapsed ALL [12]. Conversely, AP is generally not recommended for standard- or intermediate-risk ALL, solid tumors, or patients receiving monoclonal antibody monotherapy. When indicated, levofloxacin is the preferred agent for the duration of severe neutropenia (*Recommendations 2.2 and 7.1*). Evidence regarding the use of clinical tools to identify patients who may benefit from AP, such as expected duration and depth of neutropenia, MDR colonization, and predictive scores, is limited. Although pediatric data are sparse, encouraging results from adult studies have motivated experts to recommend further longitudinal and prospective research in children. A major concern remains the risk of infections caused by antibiotic-resistant bacteria, which is difficult to evaluate due to the small number of BSI observed in prospective trials, limiting the ability to draw definitive conclusions [12]. Retrospective studies exploring different AP policies across periods or centers are subject to relevant bias and confounding factors [14, 46]. Experts emphasized the importance of ASP and periodic monitoring of local ecology as key measures to guide efficient AP policies in pediatric oncology-hematology centers [47]. Additionally, although the panel addressed major issues that could inform the decision to initiate AP, other important factors, such as patient provenance from areas with high MDR prevalence or prior infections with specific pathogens, require further investigation. Finally, growing evidence on the impact of antibiotics on the gut microbiome [48, 49] and its influence on multiple clinical outcomes [50–52] should be incorporated into future consensus recommendations and RCTs.

## Conclusions

This Delphi consensus provides evidence-informed recommendations to guide the selective use of AP in pediatric oncology patients. By integrating risk stratification, screening for resistant bacteria, and stewardship principles, these statements aim to support individualized clinical decision-making while limiting unnecessary antibiotic exposure. Ongoing research and surveillance are essential to refine these strategies and to address remaining gaps, particularly regarding resistance dynamics and long-term patient outcomes.

## Supplementary Information

Supplementary Material 1

## Data Availability

The data supporting the conclusions of this article is included within the article and its additional files.

## Abbreviations

AIEOP: Associazione Italiana di Ematologia e Oncologia Pediatrica
ALL: Acute lymphoblastic leukemia
AML: Acute myeloid leukemia
ANC: Absolute neutrophil count
AP: Antibiotic prophylaxis
ASP: Antimicrobial stewardship program
BSI: Bloodstream infection
CPE: Carbapenemase-producing enterobacteriaceae
CRE: Carbapenem-resistant enterobacteriaceae
ECIL: European conference on infections in Leukemia
ESBL: Extended-spectrum β-lactamase
FQ: Fluoroquinolone
FN: Febrile neutropenia
IDSA: Infectious diseases society of America
MDR: Multidrug-resistant
MRSA: Methicillin-resistant Staphylococcus aureus
OR: Odds ratio
RCT: Randomized controlled trial
SITIP: Società Italiana di Infettivologia Pediatrica
SPOG: Swiss pediatric oncology group
VRE: Vancomycin-resistant enterococci
