# Supplementary material for: Recommendations for antibacterial prophylaxis in children receiving chemotherapy: a joint initiative of SITIP and infectious disease group of AIEOP
Source: Ital J Pediatr. 2025 Nov 25;51:309. doi: 10.1186/s13052-025-02141-1 (PMC12649064; doi:10.1186/s13052-025-02141-1)
Supplement: Supplementary file 1 — Supplementary Material 1 [file 13052_2025_2141_MOESM1_ESM.docx]

**RECOMMENDATIONS FOR ANTIBACTERIAL PROPHYLAXIS IN CHILDREN RECEIVING CHEMOTHERAPY: a joint initiative of SITIP and Infectious Disease Group of AIEOP**

Daniele Zama^1^, Davide Leardini^2,*^, Francesco Baccelli^2^, Edoardo Muratore^2^, Elio Castagnola^3^, Margherita Del Bene^4^, Maia De Luca^5^, Elisa Funiciello^6^, Federica Galaverna^5^, Riccardo Masetti^2^, Paola Muggeo^7^, Rosa Maria Mura^8^, Katia Perruccio^9^, Erica Ricci^3^, Manuela Spadea^10,11^, Andrea Lo Vecchio^4°^, Simone Cesaro^12°^

^1^ Pediatric Emergency Unit, IRCCS Azienda Ospedaliero-Universitaria di Bologna, Bologna;

^2^ Pediatric Hematology and Oncology, IRCCS Azienda Ospedaliero-Universitaria di Bologna, Bologna;

^3^ Pediatric Infectious Diseases Unit, IRCCS Istituto Giannina Gaslini, Genova, Italy;

^4^ Pediatric Infectious Disease Unit, Department of Maternal and Child Health, University Hospital "Federico II", Napoli, Italy;

^5^ Infectious Disease Unit, Bambino Gesù Children's Hospital, IRCCS, Roma, Italy;

^6^ Pediatric Infectious Diseases Unit, Regina Margherita Children's Hospital, University of Turin, Torino, Italy;

^7^ Department of Pediatric Hematology and Oncology, University Hospital of Policlinico of Bari, Bari, Italy;

^8^ Pediatric Oncology Unit, Azienda Ospedaliera Brotzu, Cagliari, Italy;

^9^ Pediatric Oncology Hematology, Mother and Child Health Department, Santa Maria della Misericordia Hospital, Perugia, Italy;

^10^ Pediatric Oncohematology, Stem Cell Transplantation and Cell Therapy Division, Regina Margherita Children's Hospital, Turin, Italy;

^11^ University of Turin, Turin, Italy;

^12^ Pediatric Hematology Oncology Unit, Department of Mother and Child, Azienda Ospedaliera Universitaria Integrata, Verona, Italy.

^*^: Corresponding author

**^°^**: these authors contributed equally to the drafting of the manuscript

**SUPPLEMENTARY MATERIAL**

**Supplementary Methods**

The Delphi process consisted of several steps. First, a survey on AP practice was shared among Italian centers treating patients with oncological diseases within the AIEOP (Associazione Italiana di Oncologia ed Ematologia Pediatrica) network. Then, a committee of experts from the AIEOP Infectious Disease Group (IDG) and SITIP (Società Italiana di Infettivologia Pediatrica) societies was established, composed of 14 experts whose expertise was demonstrated by publications, participation in other expert panels, and active participation in international scientific meetings. During the first online meeting (June 2023), the results of the survey were reviewed, and the topics of clinical interest were defined. Based on the defined topics, a literature review was performed using the Population-Intervention-Comparison-Outcome (PICO) model. Drawing from the literature evidence, a subgroup of the committee of experts wrote the statements, which were validated by the rest of the group (January 2024). After discussion and modification of the initial statements, the statements were voted on via an electronic form using a 10‐point Likert‐type scale (1 = totally disagree; 10 = totally agree), with the results of the individual votes visible to only one investigator. Consensus was defined as a total mean score higher than 7, and if this was lower a second voting would be performed after discussion of the statements.

**Supplementary Table 1:** Results of the literature research for each topic.

| **No.** |  |
| --- | --- |
| **1. What is the role of the underlying oncological diagnosis in the adoption of AP for FN?** | |
| 1.1 | The literature research strategy identified 2017 studies. After evaluation by full title and duplicates removal, 7 papers were selected as potentially appropriate to answer this clinical question and assessed by full text: 2 RCTs, and 1 observational study, 2 systematic reviews, 2 guidelines. Overall, only a few of RCTs addressed this question in pediatric ALL. |
| 1.2 | The literature research identified 1964 studies of which 8 were selected as potentially appropriate to answer this clinical question: 2 guidelines, 5 systematic reviews, and 1 RCT. |
| 1.3 |  |
| 1.4 |  |
| **2. Does the expected duration and depth of neutropenia influence the choice to perform AP?** | |
| 2.1 | The literature research strategy identified 725 studies. After evaluation by full title and duplicates removal, 10 papers were selected as potentially appropriate to answer this clinical question and assessed by full text: 3 RCTs, and 7 observational studies. The systematic review of 10 selected studies (3 RCTs, 7 observational studies) could not find indications focusing on this topic. |
| 2.2 |  |
| **3. What is the role of screening for colonization by multi-drug resistant bacteria conducted before the start of chemotherapy in the decision to perform AP?** | |
| 3.1 | The literature research strategy identified 32 studies. The literature research, after accurate revision of the full title and the abstract and duplicates removal, identified 14 studies; but only 10 of them (1 guideline, 1 systematic review, 2 RCTs and 6 observational studies) were included to answer the clinical question. |
| **4. What is the role of validated scores predictive of NF in the choice of starting AP in children with cancer?** | |
| 4.1 | The literature research strategy identified 266 studies. After evaluation by full title and duplicates removal 14 papers were identified, but no study directly addressed the role of any score on the risk of developing NF, blood-stream infections (BSI), or severe bacterial infection in patients with cancer. |
| 4.2 |  |
| **5. Is there a role for antibacterial stewardship programs and how should antimicrobial stewardship programs be applied in the decision to perform AP?** | |
| 5.1 | The literature research strategy identified 839 studies, and after evaluation by full title and duplicate removal 7 studies were assessed by full text. Of them, only 2 were included as potentially appropriate to answer this clinical question: 1 systematic review and 1 observational study. Additionally, 1 systematic review including only adult patients has been considered but not included in the study selection. |
| 5.2 |  |
| **6. What is the role of periodic assessment of local ecology in the choice of prophylactic antibiotic therapy?** | |
| 6.1 | The literature research identified 1980 studies. After accurate revision of title and abstract and duplicate removal, only 1 systematic review was selected as potentially appropriate to answer this clinical question. |
| **7. Which agents should be used for systemic AP?** | |
| 7.1 | The literature research identified 1507 studies, after accurate revision of the title and abstract, 19 studies were selected as potentially appropriate to answer this clinical question: 2 guidelines, 4 systematic reviews, 3 RCTs and 10 observational studies. |
| **8. Does systematic use of AP increase the risk of developing infections from antibiotic-resistant germs?** | |
| 8.1 | The literature research identified 1774 studies. After accurate revision of the title and abstract and duplicate removal, 9 studies were selected as potentially appropriate to answer this clinical question: 3 systematic reviews and 6 observational studies. |
